# Efficacy of different nerve block techniques with liposomal bupivacaine for postoperative analgesia in patients undergoing single-port video-assisted thoracoscopic partial lung resection

**DOI:** 10.3389/fmed.2026.1737668

**Published:** 2026-06-17

**Authors:** Ying Zhang, Dongke Liang, Guofeng Liu, Yangsi Huang, Haiping Zhong, Nuo Yang, Yu Zhong, Yanhua Chen

**Affiliations:** 1 Department of Anesthesiology, The First Affiliated Hospital of Guangxi Medical University, Nanning, Guangxi Zhuang Autonomous Region, China; 2 Department of Anesthesiology, The First People's Hospital of Yulin City, Yulin, Guangxi Zhuang Autonomous Region, China; 3 Department of Cardiothoracic Surgery, The First Affiliated Hospital of Guangxi Medical University, Nanning, Guangxi Zhuang Autonomous Region, China

**Keywords:** bupivacaine liposome, intercostal nerve block, postoperative analgesia, thoracic paravertebral nerve block, thoracoscope

## Abstract

**Objective:**

To compare postoperative analgesia efficacy between liposomal bupivacaine-administered thoracic paravertebral blockade (TPVB) and thoracoscopic direct intercostal nerve blockade (INB) in uniportal video-assisted thoracoscopic surgery (VATS) for partial lung resection.

**Methods:**

A randomized controlled design enrolled 60 elective surgery patients, randomized in a 1:1 ratio to TPVB or INB groups. TPVB received 133 mg liposomal bupivacaine (20 mL) via ultrasound-guided T4 injection; INB got the same dose via thoracoscopic-guided 3rd–7th intercostal injection. Primary endpoint: 72-h postoperative VAS scores at rest/coughing. Secondary outcomes: sufentanil use, PCIA presses, rescue analgesics, complications, and 1–6 month chest pain NRS scores.

**Results:**

Baseline demographic and clinical characteristics were comparable between the two groups. No significant differences in VAS scores at 6 h, 24 h, 48 h, or 72 h (all *p* > 0.05). There were no statistically significant differences between the two groups in intraoperative and postoperative opioid consumption, the need for rescue analgesia, postoperative recovery indicators (time to first ambulation, chest tube removal time, and length of hospital stay), or the incidence of adverse reactions. Additionally, there were no significant differences in NRS scores for chest-related chronic pain between the two groups at 1, 3, and 6 months postoperatively (*p* > 0.05).

**Conclusion:**

For uniportal VATS, liposomal bupivacaine administered via TPVB or INB showed no statistically significant differences at the primary 6-h assessment or subsequent time points in early analgesia, safety, or long-term chronic pain impact. Pain scores were low in both groups throughout the 72-h period. However, the results do not establish equivalence between the two approaches, and as this trial compared two active interventions without a placebo arm, the absolute analgesic efficacy of each technique cannot be independently determined.

**Clinical trial registration:**

https://www.chictr.org.cn/showproj.html?proj=206795, ChiCTR2300075637.

## 1 Introduction

With the widespread adoption of minimally invasive principles and advances in enhanced recovery after surgery (ERAS), uniportal video-assisted thoracoscopic surgery (VATS) for partial lung resection has become a standard surgical procedure for the treatment of early-stage lung cancer due to its advantages of minimal trauma and rapid recovery (1). Studies have shown that compared with traditional multi-port VATS, the uniportal approach has been shown to significantly reduce postoperative pain scores, shorten the length of hospital stay, and decrease the incidence of pulmonary complications (2). However, acute postoperative pain remains a key challenge affecting patient recovery. Chest wall injuries (such as intercostal nerve compression and pleural traction) and the transmission of visceral pain signals can cause moderate to severe pain, leading to restricted breathing, reduced coughing ability, and consequently increased risks of atelectasis and infection, and may even lead to chronic postsurgical pain (CPSP) (3, 4).

The clinical utility of traditional opioid-centric multimodal analgesia is constrained by side effects such as nausea, vomiting, ileus, and respiratory depression. Although NSAIDs offer an alternative, they carry risks of gastrointestinal bleeding and cardiovascular complications with prolonged use, especially in the elderly (5). Consequently, regional techniques like thoracic paravertebral block (TPVB) and intercostal nerve block (INB) have become widely adopted for thoracoscopic surgery due to their precision and ability to reduce systemic opioid consumption (6). Nonetheless, the conventional bupivacaine used in these blocks is limited by an analgesic duration of less than 24 h. Strategies to prolong its effect, such as continuous catheter infusion (with associated risks of displacement and infection) or the addition of epinephrine (which may cause tachycardia), present additional limitations (7, 8).

Liposomal bupivacaine (LB) addresses this limitation. It achieves three-phase drug release lasting up to 72 h through a multivesicular liposome delivery system, with a peak serum concentration far below the toxic threshold (2 μg/mL), which confers a favorable safety profile relative to conventional formulations (9). Studies have shown that serratus anterior plane block with a single injection of liposomal bupivacaine, compared with that with continuous catheter infusion of conventional bupivacaine, is associated with improved early postoperative recovery quality and a lower incidence of local complications. However, no significant differences were observed in postoperative pain control, lung function, or opioid consumption (8). Notably, most existing relevant studies focus on multi-port thoracoscopic procedures or explore the effects of LB combined with other block techniques (such as serratus anterior plane block). In contrast, uniportal VATS has a single operating channel, which may increase the risk of intraoperative intercostal nerve injury. This may result in differences in the mechanism of postoperative pain between patients undergoing uniportal VATS and those undergoing multi-port VATS (10–12). There remains a lack of comparative studies on the analgesic effects of LB applied in TPVB and INB for uniportal VATS.

This randomized controlled trial was designed to evaluate whether a statistically significant difference exists in postoperative analgesic efficacy between ultrasound-guided TPVB and direct-view thoracoscopic INB in patients undergoing partial lung resection via uniportal VATS. The study assessed pain scores, opioid consumption, and long-term complications to inform clinical analgesia practice.

## 2 Methods

### 2.1 Study design

This single-center, parallel-group, assessor-blinded, randomized controlled trial employed a 1:1 allocation ratio. The study protocol was registered with the Chinese Clinical Trial Registry (ChiCTR2300075637) on September 11, 2023, and approved by the Ethics Committee of The First Affiliated Hospital of Guangxi Medical University (Approval No. 2023-K232-01). Written informed consent was obtained from all participants.

An independent statistician generated the randomization sequence using an online tool (Randomizer.org) with a block size of four. Allocation results were concealed in sequentially numbered, opaque envelopes. The operator performing the nerve block opened the envelope only after patient enrollment to reveal the group assignment. Due to the nature of the interventions, the attending anesthesiologists and surgical team were aware of the group assignment during the intraoperative period. However, all postoperative outcome assessments and data analysis were conducted by investigators who were blinded to the patients’ group allocation.

### 2.2 Participants

Participants included in this study were patients aged 18–70 years, with American Society of Anesthesiologists (ASA) physical status classification I to III, body mass index (BMI) ranging from 18 to 28 kg/m^2^, and scheduled to undergo uniportal video-assisted thoracoscopic surgery for partial lung resection (including lobectomy and sublobar resection). All participants provided written informed consent prior to surgery.

Exclusion criteria included a history of chronic pain or long-term use of analgesic drugs, coagulation disorders (defined as international normalized ratio [INR] > 1.5 or platelet count [PLT] < 80 × 10^9^/L) (13), infection at the puncture site, a history of local anesthetic allergy, and severe hepatic or renal insufficiency (specifically, Child-Pugh class C or estimated glomerular filtration rate [eGFR] < 30 mL/min/1.73m^2^) (14), preoperative mental or consciousness disorders, pregnancy or lactation.

Patients were excluded post-randomization if they required conversion to thoracotomy, operative duration >4 h, or postoperative invasive mechanical ventilation >24 h.

### 2.3 Anesthesia and monitoring

Anesthesia was induced with ciprofol (0.4 mg/kg), sufentanil (0.4–0.6 μg/kg), and rocuronium (0.6 mg/kg), followed by double-lumen endobronchial intubation. Maintenance was achieved with continuous infusions of propofol (3–5 mg/kg/h), remifentanil (0.1–0.2 μg/kg/min), and rocuronium (0.1 mg/kg/h), titrated to maintain a bispectral index (BIS) of 40–60 and stable hemodynamics. The vital signs were observed and recorded before induction (T0), before nerve block (T1), 30 min after nerve block (T2), and at the end of surgery (T3).

Following induction, patients underwent invasive arterial pressure monitoring. At the conclusion of surgery, the double-lumen tube was exchanged for a single-lumen endotracheal tube, and patients were transferred to the ICU once stabilized.

For postoperative analgesia, all patients received intravenous pentazocine (30 mg) at the end of surgery. A standardized patient-controlled intravenous analgesia (PCIA) regimen was initiated, containing sufentanil (50 μg), pentazocine (60 mg), and ondansetron (16 mg) diluted to 300 mL with normal saline. The PCIA was set to a 2 mL/h background infusion, with a 6 mL bolus dose and a 15-min lockout period. Rescue analgesia was provided with intravenous flurbiprofen axetil (50 mg) for a Visual Analogue Scale (VAS) score greater than 4. If pain persisted after 30 min, oral oxycodone/acetaminophen (5 mg/325 mg) was administered. This analgesia protocol (PCIA settings, rescue analgesia criteria, and medications) was strictly standardized and applied identically to both the TPVB and INB groups.

### 2.4 Block procedure

All nerve block procedures were performed in accordance with standardized sterile techniques to ensure procedural consistency. Under sterile conditions, 10 mL of EXPAREL® (133 mg) was diluted with 10 mL of normal saline to a total volume of 20 mL (final concentration: 6.65 mg/mL) (15). All block procedures were performed after anesthesia induction and before surgical incision.

For the TPVB group: Ultrasound-guided single-point thoracic paravertebral blockade at the T4 level was performed by a designated anesthesiologist with extensive experience in ultrasound-guided regional anesthesia who was not involved in subsequent anesthesia management or data collection. Using a high-frequency linear ultrasound probe, the paravertebral space was identified lateral to the transverse process. A 22-gauge echogenic needle was advanced using an in-plane technique until the tip was positioned within the paravertebral space. After negative aspiration for blood or air, 20 mL of diluted liposomal bupivacaine was injected incrementally with gentle aspiration between aliquots. Successful block was confirmed by ultrasound visualization of pleural depression and anterior displacement (“tidal recession sign”), and by clinical assessment confirming that the surgical incision area (4th–5th intercostal space, midaxillary line) was within the expected sensory block range. This confirmation was performed by the same designated anesthesiologist immediately after injection, prior to surgical incision.

For the INB group: Under direct thoracoscopic visualization, intercostal nerve blocks were performed by the operating surgeon after thoracoscopic port placement. Using the magnification effect of the thoracoscopic display system, the anatomical locations of intercostal vessels and nerves were clearly identified. Blocks were performed at the 3rd to 7th intercostal spaces, at 5–8 cm lateral to the costovertebral junction, at the lower edge of each rib. A 22-gauge needle was advanced from the parietal pleura outward, and 4 mL of diluted liposomal bupivacaine was injected into each intercostal space, for a total volume of 20 mL (5 intercostal spaces × 4 mL). The surgeon performing the block was aware of group assignment, but was not involved in postoperative data collection or analysis.

### 2.5 Definitions of outcomes and complications

The following definitions were applied to key study outcomes and adverse events:

Patient-controlled analgesia (PCA) parameters: The PCA pump recorded both total attempts (the number of times the patient pressed the button, including attempts during the lockout interval) and effective deliveries (the number of bolus doses actually administered). The ratio of effective deliveries to total attempts was calculated for each patient as an indicator of analgesic demand relative to delivered medication.

Vascular injury: Defined as any of the following occurring during the block procedure: (1) frank blood aspiration upon needle negative pressure test, (2) visible hematoma formation on ultrasound imaging, or (3) clinical or radiological evidence of hematoma (e.g., chest radiograph or computed tomography) during the postoperative period. Minor, clinically insignificant vessel puncture that did not result in hematoma or require intervention was not classified as an adverse event unless meeting the above criteria.

Other complications: Atelectasis, pneumothorax, and local anesthetic systemic toxicity were recorded based on standard clinical and radiological criteria.

### 2.6 Study endpoints

Primary endpoint: Postoperative pain intensity assessed by VAS scores at rest and during coughing. Assessments were performed at 6, 24, 48, and 72 h postoperatively to characterize the longitudinal analgesic profile. The primary hypothesis test and corresponding statistical power (sample size calculation) were focused on the 6-h postoperative VAS score, representing the period of expected peak pain intensity.

Secondary endpoints: Analgesic-related indicators: Total sufentanil consumption, number of effective patient-controlled analgesia (PCA) presses recorded at intervals 0–6 h, 6–24 h, 24–48 h, and 48–72 h postoperatively, dosage of rescue analgesics (flurbiprofen axetil and oxycodone), and total morphine equivalent of oxycodone. Long-term pain assessment: Chest pain related to surgery was evaluated using the Numerical Rating Scale (NRS), with scores recorded at 1 month, 3 months, and 6 months postoperatively. Intra-group differences in NRS scores at 3 and 6 months were compared with baseline scores at 1 month postoperatively. Postoperative recovery quality: Assessed by recording the time to first ambulation, time to first flatus, chest tube removal time, length of hospital stay and ICU stay time. Intraoperative and postoperative adverse events: Incidence of intraoperative vascular injury; incidence of nausea, vomiting, urinary retention, and dizziness within 72 h postoperatively; and occurrence of atelectasis evaluated by postoperative chest computed tomography (CT) scan.

### 2.7 Sample size calculation

The sample size of this trial was calculated using the cough VAS score at 6 h postoperatively as the primary outcome measure. This time point was selected because postoperative pain intensity typically peaks within the first 6–12 h following thoracic surgery, representing the period of greatest analgesic demand and the most conservative estimate of effect size. Based on pilot study data (TPVB group: 2.0 ± 1.0 vs. INB group: 1.3 ± 0.6), PASS 21.0 software was used for sample size estimation (*α* = 0.05, *β* = 0.2). With a 10% attrition rate, 30 patients were required per group.

### 2.8 Statistical analysis

Statistical analyses were performed using SPSS 27.0 software. For continuous data, those conforming to a normal distribution were described as mean ± standard deviation (±s), while those not conforming to a normal distribution were expressed as median (interquartile range) [*M* (*P*_25_, *P*_75_)]. Categorical data are presented as frequencies and percentages (%). For intergroup comparisons, continuous variables were first tested for normality and homogeneity of variance. Normally distributed variables were compared using the independent samples *t*-test, while non-normally distributed variables were compared using the Mann–Whitney *U* test. Categorical variables were compared using the chi-square test or Fisher’s exact test, as appropriate.

For repeated-measures data, the appropriate analytical method was chosen according to the data distribution—repeated-measures analysis of variance (ANOVA) was used if the repeated-measures data conformed to a normal distribution and the sphericity assumption was met, while the generalized estimating equation (GEE) was adopted if the data did not conform to a normal distribution. For the primary outcome (repeated VAS measures at rest and coughing), the main inference was based on the group effect from the Generalized Estimating Equation (GEE) model with an exchangeable correlation structure. Pairwise comparisons at individual time points were descriptive only, and the corresponding *p* values are unadjusted. In the GEE analysis, the regression coefficient (*β*) was used to quantify the linear association between independent variables (group, time) and the dependent variable (VAS score). The sign of β indicated the direction of the effect, while its absolute value reflected the magnitude of the effect. The odds ratio (OR) was used to compare the relative probability of higher pain scores between groups.

## 3 Results

**Figure 1:**
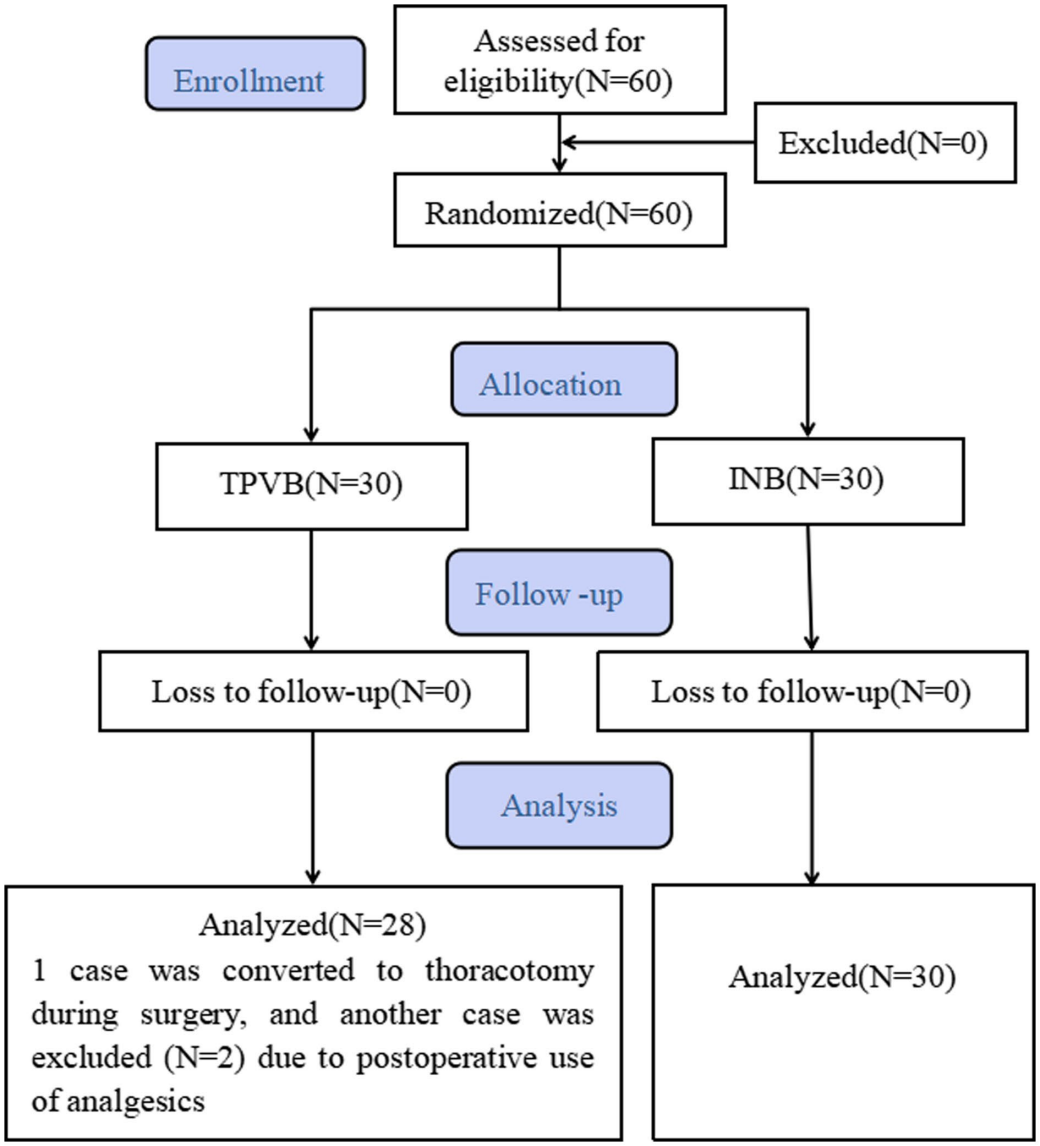
Research flow chart.

A total of 58 patients were included in the final analysis: 28 in the TPVB group and 30 in the INB group. The study analysis was conducted in accordance with the flow diagram (Figure 1). There were no significant differences in baseline demographic, surgical, and postoperative characteristics between the two groups (Table 1).

**Table 1:** Comparison of preoperative baseline data between the two groups.

	TPVB (*n* = 28)	INB (*n* = 30)	*p*
Patient characteristics			
Age (year)	52.5 ± 12.0	52.1 ± 10.1	0.881
Gender (male)	8 (28.6%)	12 (40.0%)	0.360
Body mass index (kg/m^2^)	22.8 ± 2.5	23.6 ± 3.5	0.359
ASA (I/II)	9/19 (32.1%/67.9%)	7/23(23.3%/76.7%)	0.453
Surgical characteristics			
Lobectomy	10 (35.7%)	12 (40.0%)	
Sublobar resection	18 (64.3%)	18 (60.0%)	
Operating time (min)	97.7 ± 37.1	90.0 ± 31.2	0.394
Intraoperative sufentanil (μg)	31.79 ± 8.08	32.50 ± 8.07	0.714
Intraoperative remifentanil (μg)	602.86 ± 246.26	584.97 ± 215.56	0.790
Intraoperative phenylephrine (μg)	58.57 ± 88.10	74.67 ± 116.85	0.854
Intraoperative ephedrine (mg)	1.12 ± 2.10	2.30 ± 3.81	0.345
Intraoperative atropine (mg)	0.04 ± 0.19	0.00 ± 0.00	0.140
Baseline MAP (mmHg)	94.1 ± 14.0	98.5 ± 13.7	0.232
Baseline HR	73.8 ± 8.2	76.8 ± 14.4	0.346
Baseline BIS	93.2 ± 8.3	93.9 ± 4.1	0.672
Baseline SpO_2_ (%)	100.0 (99.0, 100.0)	100.0 (99.0, 100.0)	0.911
Postoperative recovery characteristics			
First time of flatus (*d*)	2.0 (1.0, 2.0)	1.0 (1.0, 2.0)	0.073
First time out of bed (*d*)	1.0 (1.0, 1.0)	1.0 (1.0, 1.0)	0.334
Chest tube removal time (*d*)	2.0 (2.0, 2.0)	2.0 (2.0, 2.0)	0.959
Tracheal tube removal time (min)	96.5 (79.0, 128.0)	87.5 (65.0, 130.5)	0.618
Length of ICU stay (min)	160.5 (130.0, 190.0)	159.5 (127.5, 192.5)	0.580
Length of hospital stay (*d*)	2.0 (2.0, 2.0)	2.0 (2.0, 2.0)	0.762

Regarding the primary outcome, there were no statistically significant differences in VAS scores at rest or during coughing between the two groups at 6 h, 24 h, 48 h, and 72 h postoperatively (all *p* > 0.05, Table 2). The primary inferential analysis was performed using GEE. For resting VAS scores, the GEE model revealed no significant group effect (Wald *χ*^2^ = 6.56, *p* = 0.087). At the pre-specified primary 6-h time point, the odds ratio (OR) for TPVB versus INB was 2.33 (95% CI: 0.92–5.88, *p* = 0.074), indicating no statistically significant difference between groups. For cough-associated VAS scores, the GEE model similarly revealed no significant group effect (Wald *χ*^2^ = 4.22, *p* = 0.239), with an OR of 0.47 (95% CI: 0.18–1.25, *p* = 0.129) at the 6-h time point. Detailed GEE parameter estimates for all time points are presented in Table 3.

**Table 2:** Comparison of postoperative resting and cough VAS scores between the two groups of patient.

	Resting			Cough		
	TPVB	INB	*p*	TPVB	INB	*p*
Postoperative 6 h	1.0 (1.0, 1.5)	1.0 (0.0, 2.0)	0.584	3.0 (2.0, 3.5)	2.0 (2.0, 3.0)	0.538
Postoperative 24 h	1.0 (0.5, 1.0)	1.0 (0.0, 1.0)	0.695	3.0 (2.0, 4.0)	3.0 (2.0, 4.0)	0.740
Postoperative 48 h	1.0 (0.0, 1.0)	1.0 (0.0, 1.0)	0.563	3.0 (2.0, 3.0)	2.5 (2.0, 3.0)	0.974
Postoperative 72 h	1.0 (0.0, 1.0)	1.0 (0.0, 1.0)	0.892	2.0 (2.0, 3.0)	2.0 (2.0, 3.0)	0.948

*p* values for individual time points are unadjusted and presented for descriptive purposes.

**Table 3:** Estimated GEE parameters of postoperative VAS score in both groups of patients.

Outcome	Parameter	*β*	SE	Wald *χ*^2^	*p*	OR	OR95%CI	OR95%CI
VAS at rest	TPVB	0		6.564	0.087			
	TPVB at 6 h	0.845	0.473	3.189	0.074	2.327	0.921	5.882
	TPVB at 24 h	0.584	0.388	2.26	0.133	1.792	0.838	3.836
	TPVB at 48 h	0.543	0.273	3.954	0.047	1.722	1.008	2.941
	TPVB at 72 h	0				1		
	INB	0		1.622	0.654			
	INB at 6 h	0.504	0.398	1.601	0.206	1.655	0.758	3.613
	INB at 24 h	0.327	0.363	0.814	0.367	1.387	0.681	2.823
	INB at 48 h	0.136	0.209	0.426	0.514	1.146	0.761	1.724
	INB at 72 h	0				1		
VAS at cough	TPVB	0		4.220	0.239	1		
	TPVB at 6 h	−0.761	0.501	2.308	0.129	0.467	0.175	1.247
	TPVB at 24 h	0.137	0.437	0.098	0.754	1.147	0.487	2.699
	TPVB at 48 h	−0.599	0.486	1.519	0.218	0.549	0.212	1.424
	TPVB at 72 h	0				1		
	INB	0		2.438	0.487	1		
	INB at 6 h	0.484	0.510	0.903	0.342	1.623	0.598	4.405
	INB at 24 h	0.042	0.528	0.006	0.937	1.043	0.37	2.935
	INB at 48 h	0.522	0.504	1.075	0.300	1.685	0.628	4.522
	INB at 72 h	0				1		

The GEE model used an exchangeable correlation structure with the 72-h time point serving as the reference category. Within each group, odds ratios (ORs) represent the odds of higher pain scores at a given time point relative to the 72-h reference. For example, an OR of 2.327 for TPVB at 6 h indicates that the odds of higher resting VAS scores at 6 h were approximately 2.3 times the odds at 72 h within the TPVB group. The overall between-group comparison is represented by the group effect Wald test (Wald *χ*^2^ = 6.56, *p* = 0.087 for resting VAS), which assesses differences between TPVB and INB across all time points simultaneously.

For secondary outcomes, intraoperative vital sign changes were similar between the two groups, with hemodynamics maintained within a safe range and no severe hypotension or hypertension observed (Supplementary Table S1). In terms of postoperative analgesia, there were no statistically significant differences between the two groups in sufentanil consumption or the number of effective PCA presses (*p* > 0.05, see Tables 4, 5). Additionally, no statistically significant differences were found between the TPVB group and the INB group in the dosage of rescue flurbiprofen axetil, oxycodone consumption, or total morphine equivalent of oxycodone (*p* > 0.05, Table 6).

**Table 4:** Cumulative use of sufentanil in postoperative 72 h within the two groups.

	TPVB	INB	*p*
Postoperative 6 h (ml)	25.45 (9.50, 35.90)	16.00 (7.10, 27.35)	0.319
Postoperative 24 h (ml)	108.85 (86.20, 119.70)	98.55 (63.25, 111.85)	0.197
Postoperative 48 h (ml)	174.47 ± 53.13	153.09 ± 50.21	0.121
Postoperative 72 h (ml)	174.47 ± 53.13	154.69 ± 52.33	0.159

**Table 5:** Comparison of PCA pump press characteristics in the 72-h postoperative period between the two groups.

	TPVB	INB	*p*
Postoperative 6 h	0.0 (0.0, 1.0)	0.0 (0.0, 1.0)	0.812
Postoperative 6–24 h	1.0 (0.0, 2.0)	0.0 (0.0, 2.0)	0.301
Postoperative 24–48 h	0.0 (0.0, 0.0)	0.0 (0.0, 1.0)	0.088
Postoperative 48–72 h	0.0 (0.0, 0.0)	0.0 (0.0, 0.0)	0.068
Total number of analgesic pump presses	3.0 (1.0, 7.0)	1.0 (0.0, 3.5)	0.560
Ratio of effective PCA presses to total attempts	1.0 (1.0, 1.0)	1.0 (0.9, 1.0)	0.968

**Table 6:** Remedial medications and dosages in the two groups of patients during the 72 h postoperative period.

	TPVB	INB	*p*
Oxycodone (mg)	0.0 (0.0, 0.0)	0.0 (0.0, 0.0)	0.627
Flurbiprofen axetil (mg)	0.0 (0.0, 0.0)	0.0 (0.0, 0.0)	0.137
Oxycodone morphine equivalent (mg)	0.0 (0.0, 0.0)	0.0 (0.0, 0.0)	0.627

For surgery-related chest pain at 1, 3, and 6 months postoperatively, there were no statistically significant differences in NRS scores between the two groups (*p* > 0.05, Table 7). Intra-group comparisons showed that NRS scores at 3 and 6 months were significantly lower than at 1 month in the TPVB group (both *p* < 0.05), while scores at 3 months were significantly lower than at 1 month in the INB group.

**Table 7:** Postoperative 1, 3 and 6-month NRS scores of chest pain related to surgery in two groups.

	TPVB	INB	*p*
Postoperative 1 month	0.0 (0.0, 1.0)	0.5 (0.0, 1.0)	0.281
Postoperative 3 months	0.0 (0.0, 1.0)^*^	0.0 (0.0, 0.0)^*^	0.340
Postoperative 6 months	0.0 (0.0, 0.0)^*^	0.0 (0.0, 0.0)	0.997

*Comparison within the group compared with postoperative 1 month, *p* < 0.05.

Concerning intraoperative and postoperative adverse events, no statistically significant differences were found between the two groups in the incidence of nausea, vomiting, urinary retention, dizziness within 72 h postoperatively, or atelectasis indicated by postoperative chest CT (*p* > 0.05). Intraoperative vascular injury occurred in 3 cases in the TPVB group and 1 case in the INB group, with no statistically significant difference (*p* > 0.05, Table 8).

**Table 8:** Comparative analysis of postoperative adverse events between the two groups of patients [*n*(%)].

	TPVB	INB	*p*
Nausea	3 (10.7%)	3 (10%)	0.929
Vomiting	1 (3.6%)	1 (3.3%)	1
Urinary retention	1 (3.6%)	0 (0%)	0.483
Postural dizziness	8 (28.6%)	7 (23.3%)	0.649
Non-postural dizziness	2 (7.1%)	2 (6.7%)	1
Atelectasis	0 (0%)	0 (0%)	–
Vascular injury	3 (10.7%)	1 (3.3%)	0.344

## 4 Discussion

Regional nerve blocks are pivotal for post-thoracoscopic analgesia, as they prevent nociceptive signal transmission and subsequent sensitization (16). In this study, both TPVB and INB provided effective postoperative pain control following uniportal VATS, with no statistically significant difference detected at the pre-specified primary 6-h endpoint or at subsequent assessments through 72 h. VAS scores remained ≤5 throughout the observation period and declined to approximately 1 by 72 h. Several anatomical and pharmacological factors may be relevant to this observed similarity in analgesic profiles. First, the surgical incision site (4th-5th intercostal space, midaxillary to anterior axillary line) lies within the shared sensory territory of both blocks. Importantly, the dorsal rami of the spinal nerves—which innervate the paraspinal muscles and posterior dermatomes—are not involved in the surgical field of uniportal VATS. While TPVB theoretically provides more extensive blockade by anesthetizing both ventral and dorsal rami, this broader coverage confers no clinical advantage when the surgical trauma is confined to the anterolateral chest wall. The absence of dorsal rami involvement is consistent with the observation that a more proximal block (TPVB) did not outperform a more distal, targeted approach (INB) at the critical 6-h pain peak. The observed VAS reduction in both groups over 72 h may also be associated with sustained block effects concurrent with the natural resolution of surgical inflammation (16). However, we caution that the absence of statistically significant differences does not imply equivalence. The study was not designed as a non-inferiority or equivalence trial, and the sample size was powered to detect a clinically relevant difference in VAS at 6 h, but not to rule out smaller, potentially meaningful differences between techniques at later time points. Therefore, our findings should be interpreted as demonstrating comparable analgesic profiles under the current study conditions, rather than establishing equivalence.

No statistically significant differences in VAS scores were observed between the TPVB and INB groups at any time point. When interpreted against established minimal clinically important difference (MCID) benchmarks, a reduction of ≥10 mm on a 100-mm VAS or a decrease of 1–2 points for patients with moderate-to-severe pain is considered clinically meaningful (17–20). In the present study, the mean cough-associated VAS score peaked at 3 (on a 0–10 scale) at 6 h, declining to 3 at 24 h and approximately 1 by 72 h. These values fall within the mild pain range (VAS ≤ 3) for the majority of the postoperative period. Moreover, the absolute differences between groups were consistently below the MCID threshold of 1 point. Therefore, even if a statistical difference had existed, it would be unlikely to represent a clinically important advantage for either technique. Inter-individual variations in pain sensitivity and psychological factors may have contributed to the observed scores (16). Thus, the observed between-group differences are unlikely to represent clinically meaningful advantages. Furthermore, the relatively low pain scores observed in both groups may also reflect a floor effect, which could limit the ability to detect small between-group differences.

The absence of significant differences in analgesic requirements between groups is consistent with the primary outcome. Both groups showed similar effective PCA demands and total sufentanil consumption. This finding is consistent with the standardized PCA protocol applied in both groups. Standardization of the PCA protocol is associated with reduced inter-individual variability in pain perception. No significant differences were observed in intraoperative opioid consumption or postoperative rescue analgesic use. These findings are consistent with prior research: a retrospective study (21) reported no significant differences between TPVB and INB in surgical duration or postoperative VAS scores after dual-port thoracoscopic surgery, and Ma et al. (22) noted comparable postoperative pain control between the two techniques.

The lack of significant inter-group differences may be partly attributable to the use of liposomal bupivacaine in both blocks. The pharmacokinetic profile of this formulation—initial free drug release, followed by sustained low-concentration diffusion, and eventual prolonged analgesia beyond 72 h (23, 24)—could bridge potential efficacy differences between techniques. From an injection-technique perspective, single-point TPVB relies on longitudinal spread within the paravertebral space, whereas multi-level INB deposits drug directly at each targeted intercostal nerve. The prolonged duration of liposomal bupivacaine may be associated with similar clinical outcomes despite the limitations in drug distribution inherent to single-point injection. This interpretation is supported by prior safety and efficacy data: in open thoracic surgery, INB with 266 mg liposomal bupivacaine provided analgesia comparable to epidural analgesia using a similar perineural concentration (133 mg/20 mL) without serious drug-related adverse events (25). A randomized trial in total knee arthroplasty suggested that a higher dose (532 mg) may offer enhanced analgesia without increased risk (26), indicating a need for dose optimization for specific block techniques.

Anatomically, TPVB targets the highly vascularized paravertebral space, which may accelerate drug absorption (27). However, the sustained-release property of liposomal bupivacaine could counteract this effect, ensuring prolonged exposure. INB performed under direct thoracoscopic visualization lateral to the costovertebral joint (28) allows precise placement. Despite differing anatomical targets, both techniques achieved satisfactory analgesia when combined with liposomal bupivacaine, suggesting its potential as a versatile analgesic platform.

Regarding safety, vascular injury occurred in 3 of 28 patients (10.7%) in the TPVB group, compared to 1 of 28 patients (3.6%) in the INB group. All vascular injuries in the TPVB group were defined as minor vessel puncture with ultrasound-visible hematoma formation but without clinical sequelae or need for intervention; no case involved hemodynamic instability, significant blood loss, or required transfusion. Although this difference did not reach statistical significance, it may reflect the vascular density of the paravertebral space and the technical demands of ultrasound-guided TPVB (29). In contrast, INB under direct vision enabled rapid and accurate execution with minimal complications. The absence of clinically significant bleeding complications in either group supports the overall safety profile of both techniques when performed by experienced operators.

This study has several limitations. First, although the sample size was adequately powered to detect a clinically meaningful difference in the primary outcome (VAS at 6 h), it was not designed to detect smaller differences between groups. The observed VAS differences were below the MCID threshold of 1 point, supporting that no clinically meaningful advantage was present, but a larger trial would be needed to rule out smaller but potentially relevant differences. Second, the study was not powered to detect differences in low-incidence complications such as vascular injury or atelectasis. The numerically higher rate of minor vascular puncture in the TPVB group (3/28 vs. 1/28)—all clinically insignificant and requiring no intervention—should be interpreted with caution and warrants further investigation. Third, two patients were excluded after randomization because they met pre-specified exclusion criteria (conversion to thoracotomy and operative time >4 h, respectively). No postoperative outcome data were collected for these patients. Consequently, the analysis is per-protocol rather than intention-to-treat. Although the number of excluded patients is small (2/60, 3.3%) and their exclusion occurred before any outcome assessment, the possibility of attrition bias cannot be definitively excluded. In the absence of outcome data for these two participants, sensitivity analyses using imputation methods (e.g., worst-case scenario or multiple imputation) could not be meaningfully performed, as any imputed values would be entirely speculative. Therefore, while the observed results are unlikely to be materially altered by these exclusions, this limitation should be considered when interpreting the internal validity of the findings. Finally, the present analysis focused on discrete time-point VAS assessments. Alternative summary measures, such as the area under the pain intensity-time curve (cumulative pain burden), may provide a more integrated assessment of postoperative analgesic efficacy and could be considered in future studies.

## 5 Conclusion

In conclusion, for patients undergoing uniportal VATS partial lung resection, no statistically significant difference in postoperative analgesia efficacy or safety was detected between liposomal bupivacaine administered via TPVB versus INB at the pre-specified primary 6-h analysis or throughout the 72-h observation period. Pain scores remained low throughout the observation period in both groups, and no significant differences were observed in opioid consumption or adverse events. These findings suggest that both techniques may be viable options for multimodal analgesia in this surgical population. However, the results do not establish equivalence, as the study was not designed or powered as a non-inferiority trial. Furthermore, in the absence of a placebo or standard-of-care control group, the absolute analgesic efficacy of each technique cannot be independently determined from these data. A larger trial with appropriate comparators would be required to confirm the relative efficacy and establish equivalence.

## Data Availability

The original contributions presented in the study are included in the article/Supplementary material, further inquiries can be directed to the corresponding authors.
