# Supplementary material for: Efficacy of different nerve block techniques with liposomal bupivacaine for postoperative analgesia in patients undergoing single-port video-assisted thoracoscopic partial lung resection
Source: Front Med (Lausanne). 2026 Jun 17;13:1737668. doi: 10.3389/fmed.2026.1737668 (PMC13318941; doi:10.3389/fmed.2026.1737668)
Supplement: Supplementary file 1 [file Table_1.docx]

# CONSERVE Checklists

Use CONSERVE-CONSORT for completed trial reports and CONSERVE-SPIRIT for trial protocols.

| CONSERVE-CONSORT Extension: [2025/11/8] | | | | | |
| --- | --- | --- | --- | --- | --- |
| Item | Item Title | Description | | | Page No. |
| I. | Extenuating Circumstances | The conduct and reporting of this trial were not affected by any "extenuating circumstances" as defined by the CONSERVE guidelines. | | |  |
| II. | Important Modifications | 1. Since there were no "extenuating circumstances", no important or substantive modifications were made to the registered study protocol. | | |  |
|  |  | 1. Minor discrepancies between the manuscript and the registered information (detailed below) are supplementary details at the reporting level, intended to enhance the clarity of methods and the completeness of results. They do not constitute modifications to the study protocol, and thus there are no "impacts" requiring mitigation. | | | (see below) |
|  |  | 1. Not applicable. | | |  |
| III. | Responsible Parties | Not applicable. | | |  |
| IV. | Interim data | All supplementary details in the report were not based on the analysis of any interim trial data. These descriptive pieces of information were added during the manuscript writing process after the completion of data collection. | | |  |
| CONSORT Number and Item | | For each row, if important modifications occurred check “direct impact” and/or “mitigating strategy” and describe the changes in the trial manuscript or supplement. Check “no change” for items that are unaffected in the extenuating circumstance. | | | Page No. |
|  |  | No Change | Impact* | Mitigating Strategy** |  |
| 1 | Title and abstract | √ |  |  |  |
| 2 | Introduction | √ |  |  |  |
| 3 | Methods: Trial Design | √ |  |  |  |
| 4 | Methods: Participants | √ |  |  |  |
| 5 | Methods: Interventions | √ |  |  |  |
| 6 | Methods: Outcomes | √ |  |  |  |
| 7 | Methods: Sample Size | √ |  |  |  |
| 8-10 | Methods: Randomisation | √ |  |  |  |
| 11 | Methods: Blinding | √ |  |  |  |
| 12 | Methods: Statistical methods | √ |  |  |  |
| 13 | Results: Participant flow | √ |  |  |  |
| 14 | Results: Recruitment | √ |  |  |  |
| 15 | Results: Baseline data | √ |  |  |  |
| 16 | Results: Numbers analysed | √ |  |  |  |
| 17 | Results: Outcomes and estimation | √ |  |  |  |
| 18 | Results: Ancillary analyses | √ |  |  |  |
| 19 | Results: Harms | √ |  |  |  |
| 20 | Discussion: Limitations | √ |  |  |  |
| 21 | Discussion: Generalisability | √ |  |  |  |
| 23 | Other information: Registration | √ |  |  |  |
| 24 | Other information: Protocol | √ |  |  |  |
| 25 | Other information: Funding | √ |  |  |  |
| *Aspects of the trial that are directly affected or changed by the extenuating circumstance and are not under the control of investigators, sponsor or funder.  **Aspects of the trial that are modified by the study investigators, sponsor or funder to respond to the extenuating circumstance or manage the direct impacts on the trial. | | | | | |

Regarding Item 1 (Title and Abstract) and Item 5 (Interventions):

In the registration information, the group names are descriptive. In the manuscript, we used more standard and precise terminology in this field to refer to the same interventions. Specifically, we replaced "paravertebral nerve block group" with "thoracic paravertebral block (TPVB)" and adopted "intercostal nerve block (INB)" as the standard technical name for "thoracoscopic intercostal nerve block group" (the specific operation method "thoracoscopic" has been clarified in the Methods section). This constitutes the standardization and professionalization of terminology. The actual interventions received by the two groups of patients are identical to those in the registered protocol without any changes.

Regarding Item 4 (Participants):

The registered information includes the core exclusion criteria. In the manuscript, we further clarified and refined these exclusion criteria, adding "preoperative mental or consciousness disorders, pregnant women, and lactating women." This was done to ensure methodological rigor and ethical completeness without altering the core definition of participant eligibility.

Regarding Item 6 (Outcomes):

The registered information defined the primary and secondary pain outcome measures. In the manuscript, we supplemented the report with "length of ICU stay" as a routinely collected observational indicator reflecting early postoperative recovery. Additionally, we clearly described the time points for vital signs monitoring (T0-T3) to enhance methodological reproducibility. These supplements did not add new prespecified secondary outcome measures, nor did they alter the definition or analysis of any original outcomes.

Regarding Item 18 (Ancillary analyses):

In the results section, we reported "length of ICU stay" as an observational indicator. This constitutes a descriptive analysis of postoperative recovery and not a hypothesis-based supporting analysis.
