# Supplementary material for: Efficacy of different nerve block techniques with liposomal bupivacaine for postoperative analgesia in patients undergoing single-port video-assisted thoracoscopic partial lung resection
Source: Front Med (Lausanne). 2026 Jun 17;13:1737668. doi: 10.3389/fmed.2026.1737668 (PMC13318941; doi:10.3389/fmed.2026.1737668)
Supplement: Supplementary file 2 [file Table_2.docx]

Table S1. Comparison of MAP, HR, BIS andSpO_2_ measurements at each time point between the two groups

| Variable | Group | T0 | T1 | T2 | T3 |
| --- | --- | --- | --- | --- | --- |
| MAP(mmHg) | TPVB | 93.2±8.3 | 81.7±8.7 | 77.6±10.3 | 80.7±11.0 |
|  | INB | 93.9±4.1 | 83.2±12.5 | 75.8±9.0 | 85.6±11.6 |
|  | *P* | 0.672 | 0.746 | 0.496 | 0.109 |
| HR(bpm) | TPVB | 73.8±8.2 | 69.4±10.2 | 71.0±12.5 | 73.4±10.2 |
|  | INB | 76.8±14.4 | 69.4±10.2 | 75.8±15.7 | 73.7±9.8 |
|  | *P* | 0.346 | 0.746 | 0.202 | 0.897 |
| BIS | TPVB | 93.2±8.3 | 53.5±7.4 | 47.9±6.5 | 50.7±5.9 |
|  | INB | 93.9±4.1 | 50.8±7.2 | 49.0±7.0 | 49.7±6.8 |
|  | *P* | 0.672 | 0.164 | 0.534 | 0.574 |
| SpO_2_(%) | TPVB | 100.0 (99.0, 100.0) | 100.0 (100.0, 100.0) | 100.0 (99.0, 100.0) | 100.0 (100.0, 100.0) |
|  | INB | 100.0 (99.0, 100.0) | 100.0 (100.0, 100.0) | 100.0 (98.0, 100.0) | 100.0 (100.0, 100.0) |
|  | *P* | 0.911 | 0.414 | 0.506 | 0.826 |

Note: before induction of standard anesthesia (T0), before nerve block (T1), 30 minutes after completion of the nerve block (T2), and at the end of surgery (T3).
